# Multinational dietary changes and anxiety during the coronavirus pandemic-findings from Israel

**DOI:** 10.1186/s13584-021-00461-1

**Published:** 2021-03-23

**Authors:** Vered Kaufman-Shriqui, Daniela Abigail Navarro, Olga Raz, Mona Boaz

**Affiliations:** 1 Department of Nutrition Sciences, School of Health Sciences, Ariel University, Kiryat H’amada 3, 4070000 Ariel, Israel; 2 Centre for Urban Health Solutions (C-UHS), St. Michael’s Hospital, 209 Victoria St, Toronto, Canada

**Keywords:** COVID-19, Diet quality, Anxiety, Mediterranean diet score, Health surveys

## Abstract

**Background:**

Increased anxiety was frequently reported during the 2020 global COVID-19 pandemic. An association between anxiety and increased body weight has been documented. Identifying associations between diet quality and anxiety may facilitate the development of preventive dietary policy, particularly relevant since obesity appears to increase the risk of adverse COVID-19 outcomes. In this study we aim to examine associations between changes in diet pattern and body weight and anxiety levels during the COVID-19 pandemic among Israeli respondents to an international online survey.

**Methods:**

Conducted between March 30–April 252,020, this was cross-sectional, international and online study. The questionnaire was developed and tested in Hebrew and translated into six other languages: English, Arabic, Spanish, French, Italian, and Russian. The survey was conducted on a Google Survey platform, the link to which was posted on several social media platforms. Adults aged 18 or older who saw and responded to the link on a social media site comprised the study population.

**Results:**

Of the 3979 eligible respondents, 1895 indicated their current location as Israel. Most Israeli respondents completed the survey in Hebrew (83.2%) followed by Arabic (9.4%), though responses were recorded in all seven of the survey languages. The median age was 33 (IQ = 22) years, and 75.7% were female. Almost 60% indicated that their pre-pandemic diet was healthier than their current diet, and 25.2% indicated they had gained weight during the pandemic. The median Mediterranean diet score was 9 (IQ = 3). While the median General Anxiety Disorder (GAD-7) score was 5 (IQ = 8), only 37.3% of participants reported at least mild anxiety (a GAD-7 score of 5 or more), while 10.7% reported moderate anxiety or greater (a GAD-7 score of 10 or more). In a multivariate logistic regression model of at least mild anxiety, being male and completing the survey in Hebrew significantly reduced odds of at least mild anxiety, while a worsening of diet quality during the pandemic, weight gain, and isolation significantly increased odds of at least mild anxiety.

**Conclusions:**

During the COVID pandemic, changes in nutrition quality and habits were associated with greater anxiety. These findings suggest the need for routine and continuous surveillance of the nutritional and psychological consequences of outbreaks as part of healthcare preparedness efforts. Organizations responsible for community-based health services (such as Israeli health plans) should adopt specific interventions to improve case finding and support individuals at increased risk of anxiety and declining nutrition status within primary healthcare settings. These interventions should include the provision of appropriate diagnostic instruments, training of medical staff, feedback to physicians and nurses, and raising awareness among the relevant patient population and their caregivers. Primary care physicians should refer people with high anxiety or substantial weight gain during the pandemic to appropriate mental health and dietetic treatment, as needed.

**Trial registration:**

NCT04353934.

## Introduction

The global COVID-19 pandemic has created health uncertainty, especially among groups at risk for adverse COVID-19 outcomes [1]. Policies to prevent disease transmission, include containment and mitigation [2]. COVID-19 was identified in Israel during mid-March 2020. As of May 2020, and despite a relatively low case-fatality rate of 31/1,000,000, social distancing and a full lockdown was implemented in Israel [3]. The outbreak itself and the measures taken to control it have been shown to cause significant psychological distress and were associated with anxiety in China, Italy, and Spain [4–6].

Social isolation and home quarantine can produce changes in eating behaviors, food intake, and physical activity patterns, thus influencing body weight and overall health and wellbeing [7, 8]. An association between anxiety and intake of specific foods has been documented; Elevated intake of added sugars and saturated fats, but not increased calorie intake per se, has been shown to be positively associated with anxiety [9]. Limited research, especially in animal models, has demonstrated an association between anxiety and weight loss [10]. More generally, anxiety has been associated with increased emotional eating [8, 11, 12], obesity, and other risk-to-health behaviors in adults [13, 14]. At the same time, higher morbidity and mortality from COVID-19 are associated with comorbidities such as diabetes, cardiovascular disease, chronic lung disease, hypertension, and certain cancers [15]. Strategies to reduce the metabolic burden, including implementation of a balanced, healthy diet, may also protect against anxiety [16].

The Mediterranean diet (MedDiet), characterized by frequent intake of olive oil, fruits, vegetables, whole grains, legumes, fish and nuts, and infrequent intake of red and processed meats and added sugars, is associated with reduced all-cause and cause-specific mortality [9, 17]. Moreover, the MedDiet is associated with reduced risk of chronic and degenerative diseases, including certain cancers, type 2 diabetes, and cardiovascular disease [9, 18, 19]. Thus, the degree to which an individual adheres to the MedDiet can be used as an estimate of diet quality [20].

Few studies have evaluated changes in eating patterns and mental health symptoms in the general population during a pandemic [8, 21–24]. Local policies and differences in background population characteristics require tailored research to examine changes in food patterns and anxiety.

In this study, we examined whether diet quality and weight changes are associated with anxiety during the first stage of the COVID-19 outbreak in Israel.

## Methods

### Design

The present cross-sectional survey was conducted online using a convenience sample. Anxiety was assessed using the General Anxiety Disorder (GAD-7) subscale. The survey assessed the extent to which the current participant diet is similar to the MedDiet (a measure of diet quality); respondent-reported change in diet quality and body weight; and demographic characteristics.

### Ethics

The study was approved by the Institutional Ethics Board (Helsinki Committee) of Ariel University, Israel. Each participant provided informed consent prior to responding to the survey. Individuals who did not provide informed consent (indicated by clicking on the appropriate button) could not proceed with the survey.

### Study location

This international study was conducted online using a Google Survey platform. The survey was uploaded to the Ariel University Department of Nutrition Facebook page; and the r/Coronavirus community on reddit, which created a page for pandemic-associated research; and individual social media pages. Participants were encouraged to re-post the survey link on their own social media to expand exposure.

### Study population

The study population included all adult individuals who elected to complete the survey online and who provided informed consent by clicking on the appropriate button. Only the first response to the survey was included among individuals who responded more than once.

### Inclusion criteria

All adult individuals (aged 18 or older) who responded to the survey were included in any of the study languages: Arabic, English, French, Hebrew, Italian, Spanish or Russian.

### Exclusion criteria

Subjects who indicated that their age is younger than 18 years and those who did not provide informed consent were excluded. Only the index response of a given participant was included in the analysis.

### Study procedures

The survey was posted to public and personal social media pages. The survey could not be completed without first providing informed consent. Responses were automatically recorded in Google Survey.

### Survey development

The survey was translated from its original Hebrew to the following languages: Arabic, English, French, Italian, Russian, and Spanish. Each translation was performed by a native speaker of the target language who was also fluent in Hebrew. Once translated, the translation was back-translated to Hebrew by an individual bilingual in both the target language and Hebrew, thus validating the translation.

### Survey characteristics

Data were anonymous, though respondents had the option of providing an e-mail address to which information about his/her MedDiet score was mailed. The following data were elicited in the survey: 1) demographic information: age; sex; country of residence; education; work setting; current occupational status; current quarantine/isolation; health status; 2) nutrition questionnaire (MedDiet, self-reported weight change, and diet quality change) 3) anxiety questionnaire (GAD-7).

### MedDiet score

The Israeli Mediterranean diet screener (I-MEDAS) used in the current study was adapted from the original, 14-item questionnaire. The I-MEDAS predicted mortality in a prospective multi-ethnic, population-based cohort of Israeli adults. The I-MEDAS is scored such that a given question receives a value of 1 if the criterion was met, and 0 if it was not, so the total I-MEDAS score can range from 0 to 17 points [25].

### Anxiety score

The 7-item Generalized Anxiety Disorder Scale (GAD-7) was used to measure anxiety. This questionnaire is validated for use in the general populationin each of the seven languages in which the present survey was conducted [26]. The participant is asked to refer to the 2 weeks prior to the survey when responding to a set of statements. Each GAD-7 item is scored as follows: 0 (not at all), 1 (several days), 2 (more than half of the days) 3 (nearly every day). The total GAD-7 score can thus range from 0 to 21, where a higher score indicates greater anxiety [27]. Cut-off scores for mild, moderate, and severe anxiety symptoms are 5, 10, and 15, respectively [28].

### Sampling procedures

The sampling method is best described as a convenience sample with a snow-ball distribution as the survey was uploaded to both public and personal social media sites, and respondents were requested to re-post the survey on their own social media.

### Statistical analysis

Data were downloaded from Google Survey to Excel (Microsoft USA) and analyzed on SPSS v25 Statistical Analysis Software (IBM Inc.). Distributions of continuous variables were assessed for normality using the Kolmogorov-Smirnov test. All continuous data had distributions significantly deviating from normal, so they are described as median (interquartile range). Nominal variables were presented as n (%). Associations between continuous variables were described by calculating the Spearman’s correlation coefficient. Continuous variables were compared by nominal variables using the Mann-Whitney U test or the Kruskal-Wallis test as appropriate. Associations between nominal variables were assessed using the chi-square test. A multivariate logistic regression model was developed to predict the cut-point for mild anxiety or greater (GAD-7 score ≥ 5) [27, 29] using a backward stepwise approach with the log-rank test, with an entry probability of 0.05 and a removal probability of 0.1. Odds ratios with 95% confidence intervals were calculated for each covariate, and a classification table was created. All analyses were two-sided and considered significant at *p* < 0.05.

### Sample size and study power

Sample size was calculated with Raosoft® With 2144 anticipated responses, the total study was powered to provide a 95% confidence level of a population proportion and a confidence of 2%. A total of 1895 participants indicated Israel as their current location, providing 95% confidence level and a confidence limit of 2.1%.

## Results

### Database and participant dispensation

**Fig. 1:**
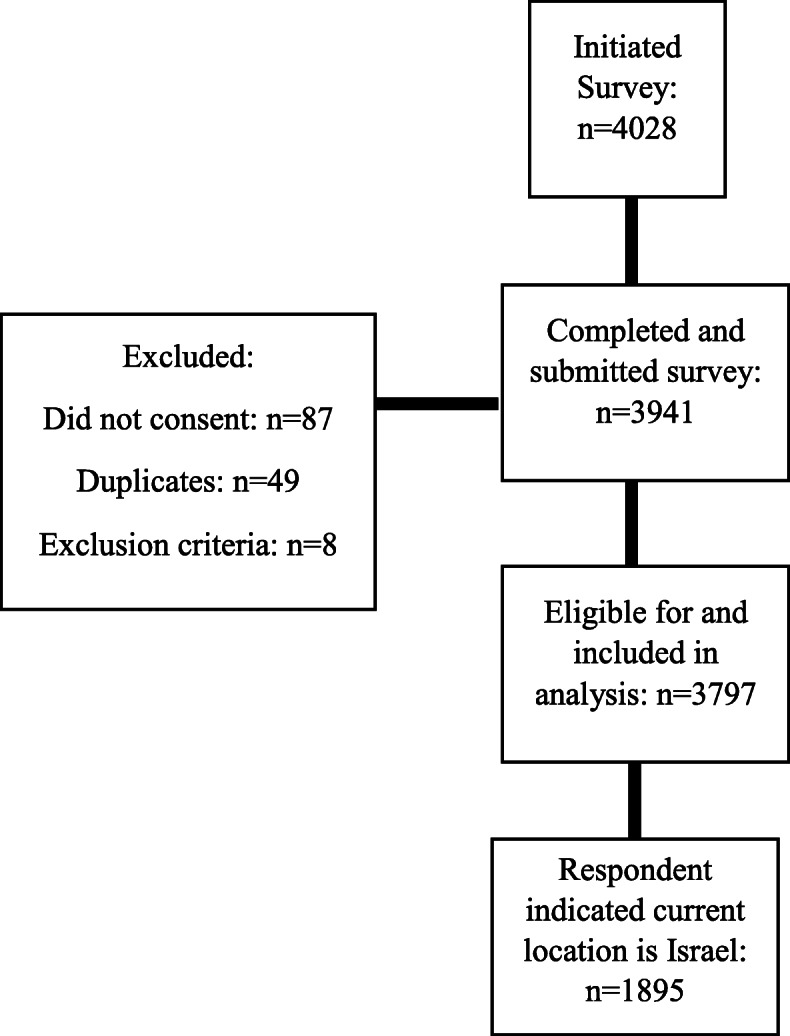
Participant Dispensation

On April 25, 22:00 Israel time, the questionnaire was closed to responses, and the database was downloaded. Participant dispensation is presented in Fig. 1. Of the 4028 individuals who initiated the survey, 3941 completed and submitted it. Omitted were 49 duplicate responses and eight respondents who indicated age younger than 18 years. A total of 3797 were included in the analysis. Of these, 1895 indicated their current location as Israel.

### Participant characteristics

Characteristics of the study population are presented in Table 1. Of the 1895 participants in the present report, more than 75% were female. Most responded to the survey in Hebrew (83.2%) or Arabic (9.4%), though responses were received in all seven of the languages into which the survey had been translated. None of the respondents had a confirmed coronavirus diagnosis at the time of completing the survey, but 17 were suspected cases without laboratory confirmation, and one was recovering from the virus. Most participants were currently employed and presently working. More than 60% held a bachelor’s degree or higher. Of the 1435 female participants, fewer than 4% were pregnant or within 6 months of delivery.

**Table 1:** Study Population Characteristics

Characteristic	Population Value
Age (years, median (interquartile range))	33 (22)
Sex n (% female)	1435 (75.7)
Reproductive Status (Females only) n (%)	
Neither pregnant nor 6 months post delivery	1060 (73.9)
Six months or less post delivery	35 (2.4)
Currently pregnant	22 (1.5)
Language n (%)	
Arabic	179 (9.4)
English	87 (4.6)
French	2 (0.1)
Hebrew	1576 (83.2)
Italian	24 (1.3)
Russian	10 (0.5)
Spanish	17 (0.9)
Health Status n (%)	
Healthy	1839 (97.0)
Sick with coronavirus	0
Suspected of coronavirus	17 (0.9)
Recovering from coronavirus	1 (0.1)
Sick but not with coronavirus	38 (2.1)
Smoking Status n (%)	
Never smoked	1307 (69.0)
Former smoker	338 (17.8)
Occasional smoker	88 (4.6)
Smokes at least one cigarette/day	161 (8.5)
Usual Place of employment n (%)	
Large/medium private sector company	333 (17.6)
Small private sector company	260 (13.7)
Public sector/non-profit	685 (36.1)
Pensioner/unemployed	378 (19.9)
Independent business owner/freelancer	224 (11.8)
Other	15 (0.8)
Work Status During Pandemic n (%)	
Leave of absence with pay	126 (6.6)
Leave of absence without pay	485 (25.6)
Retired/Pensioner	46 (2.4)
Still Working	886 (46.8)
Unemployed	131 (6.9)
Other	221 (11.7)
Education n (%)	
Fewer than 12 years	18 (0.9)
High school diploma/matriculation	486 (25.6)
Professional license (technician, tradesperson, etc.)	131 (6.9)
Bachelor’s degree	643 (33.9)
Master’s degree or higher	601 (31.7)
Other	16 (0.8)

### Lifestyle and dietary characteristics

Table 2 displays the lifestyle and dietary characteristics of the study population. Respondents were asked to rate the quality of their diets during the pandemic in relation to their usual diets. Most participants (55%) indicated that their pre-pandemic diet was healthier than their current diet. Approximately 1/3 of respondents did not detect a difference in their diets before vs. after the outbreak, while 13% felt their diet had improved during the pandemic. Respondents who reported a worsening of diet quality had significantly lower MedDiet scores: 9 (3) vs. 10 (3), *p* < 0.0001. A greater proportion of participants who reported a worsening of diet quality also reported weight gain compared to those who did not report a worsening of diet quality (36.7% vs. 8.3%, *p* < 0.0001).

**Table 2:** Lifestyle and Dietary Characteristics of the Study Population

Characteristic	Population Value
Minutes of exercise/week prior to pandemic (median (interquartile range))	60 (147)
Minutes of exercise/week in the past week (median (min-max))	30 (120)
Weight change since start of coronavirus pandemic n (%)	
Yes, weight gain	477 (25.2)
Yes, weight loss	227 (12.0)
No	668 (35.3)
I don’t know	521 (27.5)
Quantity of weight change among those reporting change	
Weight gained (median (interquartile range))	2 (1.5)
Weight lost (median (interquartile range))	-2 (2)
Vegan/Vegetarian n (%)	193 (10.2)
Takes nutrition supplements n (%)	406 (21.4)
MedDiet Score (median (interquartile range))	9 (3)
Change in diet quality n (%)	
Healthier prior to pandemic	1043 (55.0)
No difference	599 (31.6)
Healthier with pandemic	246 (13.0)
No response	7 (0.4)

No weight change was reported by 35.3% of respondents, and 27.5% did not know if weight change had occurred during the lockdown. Of 704 (37.2%) participants who did report weight change, most (67.8%) reported weight gain while the rest reported weight loss. Median weight change was 2 kg, whether individuals gained or lost weight.

Weight gain was significantly, inversely, associated with the change in diet quality. While the median MedDiet score was 9 (3) among participants who gained weight and among those who did not, the difference was significant by group (*p* = 0.002), and perceptible via mean values:8.92 ± 2.21 vs. 9.33 ± 2.37. Among people who gained weight, 84.5% indicated that their pre-outbreak diets were healthier than their current diets. By contrast, 15.5% of participants who reported no weight change or weight loss indicated that their pre-pandemic diets were healthier, *p* < 0.0001.

A 50% relative reduction in time devoted to exercise was observed during the pandemic, declining from a median of 60 min per week to 30 min per week, p < 0.0001. The amount of time reported spent exercising prior to the outbreak did not differ by weight change status, and was a median 60 (147) in both people who gained and those who did not gain weight (*p* = 0.72). Respondents who gained weight in the course of the week prior to the survey (during the pandemic lockdown) reported 30 (120) minutes of exercise/week, while those who did not gain weight reported 40 (118) minutes of exercise/week, *p* = 0.002.

Similarly, time spent exercising prior to the pandemic did not differ by a subsequent change in diet quality and was 60 (147) minutes/week in people whose diet worsened as well as among those whose diet did not worsen during the outbreak (*p* = 0.62). After the outbreak, people whose diet worsened reported 30 (102.5) minutes/week of exercise, compared to 60 (147) minutes/week among those whose diets did not worsen, *p* < 0.0001.

### Anxiety

Measures of anxiety and emotional stress are shown in Table 3. The population score was a median of 4 (6), indicating anxiety levels lower than mild. Isolation was rated on a 10-point scale at a median of 2 (3) in general, increasing to a median score of 3 (5) after the crisis, *p* < 0.0001. Median values for the number of people to which the respondent could turn to for urgent help appear similar before vs. during the coronavirus pandemic: (5 (1) vs. 5 (2); the difference is significant (*p* < 0.0001), and the difference is apparent when examining mean values: 4.3 ± 1.2 prior to and 4.1 ± 1.3.

**Table 3:** Anxiety Measures

Characteristic	Population Value
GAD-7 score (median (interquartile range))^a^	
Feeling nervous, anxious or on edge	1 (1)
Not being able to stop or control worrying	0 (1)
Worrying too much about different things	1 (1)
Trouble relaxing	0 (1)
Being so restless that it is hard to sit still	0 (1)
Becoming easily annoyed or irritable	1 (1)
Feeling afraid as if something terrible might happen	0 (1)
Total GAD-7 Score (median (interquartile range))	4 (6)
How isolated do you feel in general?^b^ (median (interquartile range)	2 (3)
How isolated do you feel in the current situation (during the pandemic)?^b^ (median (interquartile range)	3 (5)
In general, if you needed to borrow $20, or a ride to the doctor’s office, or any other urgent assistance, to how many people could you turn?^c^ (median (interquartile range)	5 (1)
In the current situation, if you needed to borrow $20, or a ride to the doctor’s office, or any other urgent assistance, to how many people could you turn?^c^ (median (interquartile range)	5 (2)

^a^The GAD-7 scale asks the respondent to refers to the 2 weeks prior to the survey. Each item on the scale can receive is scored as follows: 0 (not at all), 1 (several days), 2 (more than half of the days) 3 (nearly every day); thus, the total score can receive a value from 0 to 21, where a higher score indicates greater anxiety

^b^The question about isolation requested the participant to respond on a 10-point Likert scale where 1 = very little and 10-very much

^c^The question requested the participant to respond on a 7 point Likert scale where 0 = no one and 6 = more than 5 people

### Associations between anxiety and diet quality and weight gain

Among individuals reporting that their diet was healthier prior to the coronavirus pandemic, the median total GAD-7 score was 4 (5), compared to 2 (5) among those who reported no change or an improvement in diet quality since the start of the outbreak, *p* < 0.0001. Similarly, the GAD-7 score was significantly higher in individuals who reported weight gain during the pandemic vs. those who did not report weight gain: 5 (6) vs. 3 (5), *p* < 0.0001. Consistent with this, the GAD-7 score was significantly, positively associated with weight change, rho = 0.207, *p* < 0.0001. The GAD-7 score was also positively associated with the degree to which the respondent felt isolated: rho = 0.331, *p* < 0.0001. The amount of exercise reported by the respondent was weakly, inversely associated with the GAD-7: rho = − 0.051, *p* = 0.029.

### Anxiety and respondent characteristics

The GAD-7 score was not significantly associated with age. Table 4 displays the GAD-7 score by participant characteristics. GAD-7 scores were significantly higher among women than men. Among women, anxiety scores did not differ by reproductive status. Anxiety also differed significantly by the language in which the survey was completed, such that those who completed the survey in Hebrew reported the lowest median scores. Anxiety scores did not differ by health status, smoking status, vegetarian/vegan diet, or usual place of employment. GAD-7 scores differed by current employment status and were highest among the currently unemployed. Respondents with 12 years of formal education had higher anxiety scores than others.

**Table 4:** GAD-7 Scores by Study Population Characteristics

Characteristic	GAD-7 Score Median (interquartile range)	***p***-value
**Sex**		**< 0.0001**
**Female**	**4 (6)**	
**Male**	**2 (4)**	
**Reproductive Status (Females only)**		**0.738**
**Neither pregnant nor six months post delivery**	**4 (6)**	
**Six months or less post delivery**	**4 (5)**	
**Currently pregnant**	**4 (6.5)**	
**Language**		**< 0.0001**
**Arabic**	**6 (5)**	
**English**	**4 (5)**	
**French**	**4.5 (−)**	
**Hebrew**	**3 (5)**	
**Italian**	**7 (5)**	
**Russian**	**6 (7)**	
**Spanish**	**7 (7)**	
**Health Status**		**0.07**
**Healthy**	**3 (5)**	
**Sick with coronavirus**	**–**	
**Suspected of coronavirus**	**6 (3.5)**	
**Recovering from coronavirus**	**–**	
**Sick but not with coronavirus**	**4 (6.5)**	
**Smoking Status**		**0.337**
**Never smoked**	**3 (5)**	
**Former smoker**	**3 (5)**	
**Occasional smoker**	**4 (5.25)**	
**Smokes at least one cigarette/day**	**4 (6)**	
**Usual Place of employment**		**0.452**
**Large/medium private sector company**	**3 (7)**	
**Small private sector company**	**4 (10)**	
**Public sector/non-profit**	**4 (5.5)**	
**Pensioner/unemployed**	**4 (6.5)**	
**Independent business owner/freelancer**	**3 (6.5)**	
**Other**	**3 (8)**	
**Work Status During Pandemic**		**< 0.0001**
**Leave of absence with pay**	**3 (5)**	
**Leave of absence without pay**	**3 (5)**	
**Retired/Pensioner**	**3 (5.5)**	
**Still Working**	**3 (5)**	
**Unemployed**	**6 (5.5)**	
**Other**	**3 (5)**	
**Education**		**0.008**
**Fewer than 12 years**	**5 (8.25)**	
**High school diploma/matriculation**	**3 (5)**	
**Professional license (technician, tradesperson, etc.)**	**4 (6)**	
**Bachelor’s degree**	**3 (5)**	
**Master’s degree or higher**	**3 (5)**	
**Other**	**4 (17.25)**	

Table 5 presents the multivariate logistic regression model of at least a mild anxiety (GAD-7 score of 5 or greater). The following variables were entered into the regression model: age, sex, the language in which the questionnaire was completed, current isolation score, duration of physical activity during the COVID-19 outbreak (minutes/week), education, change in diet pattern (worsening yes/no), weight gain (yes/no), smoking status (current smoker yes/no) and MedDiet score. The final model was arrived at using a backward, stepwise approach and did not include smoking status, education, weekly minutes of physical activity during the outbreak. Though age was not significantly associated with the outcome, it was retained in the model as a universal covariate. The model was significant and correctly classified 68.9% of study participants for at least mild anxiety. As can be seen, each 1-point increase in current isolation score increased odds of at least mild anxiety by 19%. Male sex reduced the odds of at least mild anxiety by almost 60%. Individuals who reported that their current diets were less healthy than their diets prior to the COVID-19 pandemic had an almost 60% increase in odds of at least mild anxiety, and those who reported weight gain had increased odds of the anxiety of more than 46%. Completing the survey in Hebrew vs. any other language reduced the odds of mild anxiety by almost 43%.

**Table 5:** Multivariate Logistic Regression Model of Moderate to Severe Anxiety (GAD-7 score ≥ 5)

Variable	Odds Ratio	95% Confidence Interval	*p*-value
Current Isolation Score (1–10)	1.191	1.145–1.238	< 0.0001
Sex (M = 1)	0.413	0.320–0.534	< 0.0001
Language (Hebrew vs. any other language)	0.572	0.435–0.752	< 0.0001
Change in diet (diet worse = 1)	1.626	1.307–2.024	0.002
Weight gain (yes = 1)	1.467	1.150–1.879	< 0.0001
Age (years)	1.00	0.993–1.008	0.944
Constant	0.402		< 0.0001

Female sex (vs. male) was the indicator variables for sex; any college degree (bachelor’s degree or higher = 1 (vs. any other response) and was the indicator variable for education; Spanish was the indicator variable for language (vs. any other language); reporting that the current diet was worse than the pre-pandemic diet = 1 (vs. no change or improvement in diet = 0) and was the indicator for change in diet

## Discussion

The present study indicates that diet quality was inversely associated with anxiety during the 2020 COVID-19 pandemic: every one-point increase in the MedDiet score was associated with an almost 7% decrease in the odds of moderate to severe anxiety. More than 50% of study participants had a GAD-7 score consistent with at least mild anxiety. This rate is considerably higher than the prevalence of anxiety in the general population, which is estimated to be 1.9–5.1% [30].

High levels of anxiety were reported in other countries during the first outbreak stages, using online cross-sectional surveys. A study among the Chinese population assessed anxiety using the GAD-7 questionnaires and reported on 35.1% with moderate anxiety [21]. A survey among Italian adults using the State-Trait Anxiety Inventory (STAI-Y) reported on 37.19% who were defined in a higher state of anxiety [31]. In a survey conducted in Spain, during the initial stages of the pandemic, anxiety symptoms that were measured using the Depression, Anxiety, and Stress Scales (DASS-21) reported on 25.0% anxiety symptoms [32] while another study which used the GAD-7 reported on 12.3% with moderate to severe anxiety symptoms [22]. Evidence examining factors associated with anxiety due to the COVID-19 pandemic in Israel are limited. A recently published study suggested that increased psychological distress was more common among younger women with pre-existing chronic conditions [33], higher anxiety rates were reported among pregnant women [34]. Worsening of income loss was associated with exacerbation of depression was reported in additional study [35], at the same manner in a survey conducted among 1500 Jewish respondents aged 20–65 a high percentage of respondents reported an increase in depression compared with the regular pre-COVID situation [24]. Although differences in anxiety measures and differences in coping strategies across countries preclude a direct comparison, the relatively high level of anxiety in our study may reflect that anxiety was measured during a nation-wide total lockdown. Findings from in-depth interviews indicate that among those experiencing higher anxiety, the lockdown has been associated with more significant challenges such as lack of space in the home environment, shortage in access to supplies, lack of savings, and loss of income [36].

It has been shown that metabolic status (including obesity), age, and sex influence the clinical severity of COVID-19 [37]. Poor diet quality (which may foreshadow obesity) age and sex were associated with both diet quality and anxiety in the present study. A higher MedDiet score was also associated with less self-reported weight gain and an improved diet pattern during the pandemic. Increased MedDiet score was associated with reduced odds of moderate to severe anxiety, while a decline in diet quality during the outbreak compared to the pre-pandemic diet increased risk for this outcome, even after controlling for key explanatory variables. Our findings are in agreement with results from Mediterranean countries. In a recent study conducted in Greece and Spain, higher anxiety scores (as measured by the GAD-7) were associated with lower MedDiet scores [22]. In addition, adverse eating behaviors, such as restrained and emotional eating, were associated with higher symptoms of depression and anxiety. Findings from a recent international survey indicate that the COVID-19 related quarantine, was associated with overeating and consuming food of a poorer quality [8], similar findings were reported in an Israeli survey in which concern about deterioration in their financial situation, poor health, and gender (women) predicted a significant increase in the amount of food intake [24]. Additionally, a study from Poland reported on either weight loss and weight gain among adults during lockdown [38].

Israel has an efficient primary care healthcare system, providing a broad basket of services [39], which can improve the identification of patients with anxiety at times of crisis. Since the mental health reform in July 2015, Israeli citizens and permanent residents are entitled to receive mental health care services through their chosen health fund, with the costs of care covered by the funds [40]. Mental health issues carry a stigma, which may lead to avoidance of help-seeking since primary care physicians participate in the referral process [41]. However, a previous study in a national representative sample of adult Israelis has shown gender differences in the use of mental health services, with women being more likely to seek mental health help regardless of socioeconomic or morbidity [42]. In the current study, female sex emerged as a significant risk factor for anxiety. Therefore, the first step could be anxiety screening through the primary healthcare system, which could make this a national priority during the pandemic. This would encourage the four Israeli health plans to approach this disease like any other and use standard disease management tools. Additionally, since we found that worsening of diet quality during the pandemic and weight gain doubled the odds for higher anxiety, we suggest that physicians will consider referring people with high anxiety or weight gain during the pandemic to dietitians. Furthermore, dietitians in healthcare should be informed on those associations and alert primary health care physicians in case of concern.

Therefore, steps should be taken to improve case-finding of people with higher anxiety among primary physicians and those with adverse eating behaviors among dietitians. This requires the provision of appropriate diagnostic instruments, training of medical staff, feedback to physicians and nurses, and raising awareness among the relevant patient population and their caregivers.

Findings herein must be considered in the framework of study limitations. The data are cross-sectional. Associations between diet quality and anxiety are correlational only. Because the exposure (diet quality) and outcome (anxiety score) were measured simultaneously, and, as such, causality cannot be inferred.

## Conclusions

In summary, this online survey conducted in a variety of widely-spoken languages in Israel demonstrates a high frequency of anxiety symptoms in Israel and an inverse association between diet quality and anxiety level. Poor diet quality was associated with weight gain, which could contribute to obesity. These results could inform the development of new preventive strategies aimed at bettering diet quality and eating behaviours and mental health problems during the COVID-19 pandemic. Further prospective studies are needed to confirm our findings in different populations with different degrees of COVID-19 pandemic severity.

## Data Availability

Data collected for this study includes individual participants’ data. Data cannot be publicly accessible due to Institutional Review Board guidelines. We are open to collaborations with other researchers upon contacting us.

## Abbreviations

COVID-19: Coronavirus Disease 2019
DASS− 21: Depression, Anxiety, and Stress Scales
GAD-7: General Anxiety Disorder
I-MEDAS: Israel-Mediterranean Diet Adherence Screener
MedDiet: Mediterranean diet
STAI-Y: State-Trait Anxiety Inventory

## References

[1] Lauri Korajlija A, Jokic-Begic N (2020). COVID-19: concerns and behaviours in Croatia. Br J Health Psychol.

[2] Flattening the COVID-19 peak: Containment and mitigation policies [http://www.oecd.org/coronavirus/policy-responses/flattening-the-covid-19-peak-containment-and-mitigation-policies-e96a4226/].

[3] Clarfield AM, Dwolatzky T, Brill S, Press Y, Glick S, Shvartzman P, Doron II (2020). Israel ad hoc COVID-19 committee: guidelines for Care of Older Persons during a pandemic. J Am Geriatr Soc.

[4] Wang C, Pan R, Wan X, Tan Y, Xu L, Ho CS, Ho RC (2020). Immediate psychological responses and associated factors during the initial stage of the 2019 coronavirus disease (COVID-19) epidemic among the general population in China. Int J Environ Res Public Health.

[5] Qiu J, Shen B, Zhao M, Wang Z, Xie B, Xu Y (2020). A nationwide survey of psychological distress among Chinese people in the COVID-19 epidemic: implications and policy recommendations. Gen Psychiatr.

[6] Shatri H, Faisal E, Putranto R (2020). Mass panic disaster management in COVID-19 pandemic. Acta Med Indones.

[7] Naja F, Hamadeh R (2020). Nutrition amid the COVID-19 pandemic: a multi-level framework for action. Eur J Clin Nutr.

[8] Ammar A, Brach M, Trabelsi K, Chtourou H, Boukhris O, Masmoudi L, Bouaziz B, Bentlage E, How D, Ahmed M (2020). Effects of COVID-19 home confinement on eating behaviour and physical activity: results of the ECLB-COVID19 international online survey. Nutrients.

[9] Zaragoza-Martí A, Cabañero-Martínez MJ, Hurtado-Sánchez JA, Laguna-Pérez A, Ferrer-Cascales R (2018). Evaluation of Mediterranean diet adherence scores: a systematic review. BMJ Open.

[10] Morris A (2019). Anxiety-induced weight loss. Nat Rev Endocrinol.

[11] Yönder Ertem M, Karakaş M (2020). Relationship between emotional eating and coping with stress of nursing students. Perspect Psychiatr Care.

[12] Ulrich-Lai YM, Fulton S, Wilson M, Petrovich G, Rinaman L (2015). Stress exposure, food intake and emotional state. Stress.

[13] Strine TW, Mokdad AH, Dube SR, Balluz LS, Gonzalez O, Berry JT, Manderscheid R, Kroenke K (2008). The association of depression and anxiety with obesity and unhealthy behaviors among community-dwelling US adults. Gen Hosp Psychiatry.

[14] Masana MF, Tyrovolas S, Kolia N, Chrysohoou C, Skoumas J, Haro JM, Tousoulis D, Papageorgiou C, Pitsavos C, Panagiotakos DB (2019). Dietary patterns and their association with anxiety symptoms among older adults: the ATTICA study. Nutrients.

[15] Porzionato A, Emmi A, Barbon S, Boscolo-Berto R, Stecco C, Stocco E, Macchi V, De Caro R (2020). Sympathetic activation: a potential link between comorbidities and COVID-19. FEBS J.

[16] Wu S, Fisher-Hoch SP, Reininger BM, McCormick JB (2018). Association between fruit and vegetable intake and symptoms of mental health conditions in Mexican Americans. Health Psychol.

[17] de Koning L, Anand SS (2003). Adherence to a Mediterranean diet and survival in a Greek population. Trichopoulou A, Costacou T, Bamia C, Trichopoulos D. N Engl J Med.

[18] Dernini S, Berry EM, Serra-Majem L, La Vecchia C, Capone R, Medina FX, Aranceta-Bartrina J, Belahsen R, Burlingame B, Calabrese G (2017). Med diet 4.0: the Mediterranean diet with four sustainable benefits. Public Health Nutr.

[19] Schwingshackl L, Schwedhelm C, Galbete C, Hoffmann G (2017). Adherence to Mediterranean diet and risk of Cancer: an updated systematic review and meta-analysis. Nutrients.

[20] Gil Á, Martinez de Victoria E, Olza J (2015). Indicators for the evaluation of diet quality. Nutr Hosp.

[21] Huang Y, Zhao N (2020). Generalized anxiety disorder, depressive symptoms and sleep quality during COVID-19 outbreak in China: a web-based cross-sectional survey. Psychiatry Res.

[22] Papandreou C, Arija V, Aretouli E, Tsilidis KK, Bulló M (2020). Comparing eating behaviours, and symptoms of depression and anxiety between Spain and Greece during the COVID-19 outbreak: cross-sectional analysis of two different confinement strategies. Eur Eat Disord Rev.

[23] Kaya S, Uzdil Z, Çakroğlu FP (2020). Evaluation of the effects of fear and anxiety on nutrition during the COVID-19 pandemic in Turkey. Public Health Nutr.

[24] Laron M, Goldwag R, Hartal M (2020). Predictors of health behaviors during the COVID-19 pandemic and preferences regarding receipt of professional services. In.

[25] Abu-Saad K, Endevelt R, Goldsmith R, Shimony T, Nitsan L, Shahar DR, Keinan-Boker L, Ziv A, Kalter-Leibovici O (2019). Adaptation and predictive utility of a Mediterranean diet screener score. Clin Nutr.

[26] Löwe B, Decker O, Müller S, Brähler E, Schellberg D, Herzog W, Herzberg PY (2008). Validation and standardization of the generalized anxiety disorder screener (GAD-7) in the general population. Med Care.

[27] Spitzer RL, Kroenke K, Williams JB, Löwe B (2006). A brief measure for assessing generalized anxiety disorder: the GAD-7. Arch Intern Med.

[28] Kroenke K, Spitzer RL, Williams JB, Monahan PO, Löwe B (2007). Anxiety disorders in primary care: prevalence, impairment, comorbidity, and detection. Ann Intern Med.

[29] Kroenke K, Outcalt S, Krebs E, Bair MJ, Wu J, Chumbler N (2013). Yu Z: association between anxiety, health-related quality of life and functional impairment in primary care patients with chronic pain. Gen Hosp Psychiatry.

[30] Wittchen HU (2002). Generalized anxiety disorder: prevalence, burden, and cost to society. Depress Anxiety.

[31] Forte G, Favieri F, Tambelli R, Casagrande M (2020). The enemy which sealed the world: effects of COVID-19 diffusion on the psychological state of the Italian population. J Clin Med.

[32] Rodríguez-Rey R, Garrido-Hernansaiz H, Collado S (2020). Psychological impact and associated factors during the initial stage of the coronavirus (COVID-19) pandemic among the general population in Spain. Front Psychol.

[33] Horesh D (2020). Kapel Lev-Ari R, Hasson-Ohayon I: risk factors for psychological distress during the COVID-19 pandemic in Israel: loneliness, age, gender, and health status play an important role. Br J Health Psychol.

[34] Taubman-Ben-Ari O, Chasson M, Abu Sharkia S, Weiss E (2020). Distress and anxiety associated with COVID-19 among Jewish and Arab pregnant women in Israel. J Reprod Infant Psychol.

[35] Hertz-Palmor N, Moore TM, Gothelf D, DiDomenico GE, Dekel I, Greenberg DM, Brown LA, Matalon N, Visoki E, White LK, et al. Association among income loss, financial strain and depressive symptoms during COVID-19: evidence from two longitudinal studies. medRxiv. 2020. 10.1101/2020.09.15.20195339. 10.1016/j.jad.2021.04.054 PMC8460400 34022550

[36] Nyashanu M, Simbanegavi P, Gibson L (2020). Exploring the impact of COVID-19 pandemic lockdown on informal settlements in Tshwane Gauteng Province, South Africa. Glob Public Health.

[37] Weiss P, Murdoch DR (2020). Clinical course and mortality risk of severe COVID-19. Lancet.

[38] Sidor A, Rzymski P (2020). Dietary choices and habits during COVID-19 lockdown: experience from Poland. Nutrients.

[39] Porath A, Lev B (1995). The new Israeli national health insurance law and quality of care. Int J Qual Health Care.

[40] Rosen B, Waitzberg R, Merkur S (2015). Israel: health system review. Health Syst Transit.

[41] Ben Natan M, Drori T, Hochman O (2017). The impact of mental health reform on mental illness stigmas in Israel. Arch Psychiatr Nurs.

[42] Levinson D, Ifrah A (2010). The robustness of the gender effect on help seeking for mental health needs in three subcultures in Israel. Soc Psychiatry Psychiatr Epidemiol.

